# Evaluation of tumor localization accuracy on fast ring-gantry cone-beam computed tomography using patient-specific breathing curves and a dynamic anthropomorphic thorax phantom^☆^

**DOI:** 10.1016/j.phro.2026.101014

**Published:** 2026-06-03

**Authors:** Lars H.B.A. Daenen, Didier Lustermans, Tim H.A. Stassen, Juliane Szkitsak, Roua Abdulrahim, Jo Goossens, Gina Rishmawi, Richard Canters, Ilaria Rinaldi, Frank Verhaegen, Gabriel Paiva Fonseca

**Affiliations:** a Department of Radiation Oncology (MAASTRO), GROW Research Institute for Oncology and Reproduction, Maastricht University Medical Centre+, Maastricht, the Netherlands; b Department of Radiation Oncology, Universitätsklinikum Erlangen, Friedrich-Alexander-Universität Erlangen-Nürnberg, Erlangen, Germany; c Research Group NuTeC, Centre for Environmental Sciences, Hasselt University, Diepenbeek, Belgium; d Iridium Kankernetwerk, University of Antwerp, Antwerp, Belgium

**Keywords:** HyperSight CBCT, Lung cancer, Radiotherapy, Respiratory tumor motion, Tumor delineation, 4DCT

## Abstract

**Background and purpose:**

Fast cone beam computed tomography (CBCT) on ring-gantry systems allows for improved image quality and fast 6-second acquisition. However, 6-second acquisition might pose challenges regarding target delineation and capturing the full range of motion of moving lung tumors. Especially, in patients with slow (period > 6 s) or irregular breathing, capturing the entire tumor motion might not be guaranteed.

**Materials and methods:**

This study evaluated localization and volumetric accuracy of 6- and 60-second CBCT scans in an in-house dynamic anthropomorphic thorax phantom, with synchronized imaging capabilities. The phantom was scanned for a sinusoidal and four patient-derived breathing patterns, including regular, slow or irregular breathing. Target position and volume for 6- and 60-second acquisitions were compared to ground truth delineation on time-averaged 4D computed tomography (4DCT) reconstruction, assessing if 6-second acquisition is sufficient to accurately capture the tumor motion.

**Results:**

For sinusoidal and regular patient-derived motion, both 6- and 60-second CBCT acquisition captured target motion, compared to 4DCT (Dice Similarity Coefficient, DSC > 0.9). For large amplitudes, only one out of three 6-second scans fully captured target motion (DSC > 0.85). For slow and irregular patient-derived patterns, localization errors and volume differences up to 10.3 mm and 119% were observed using 6-second acquisition, compared to 4DCT, with superior localization and volumetric accuracy of the 60-second acquisition.

**Conclusion:**

The 6-second protocol showed accurate results, capturing full target motion for regular breathing patterns. Adaptive protocols, taking into account patient-specific breathing periods and irregularities may be preferred in patients exhibiting slow or irregular breathing.

## Highlights

• An anthropomorphic dynamic thorax phantom simulated patient-specific breathing.
• Fast cone-beam computed tomography (CBCT) captured full regular breathing motion.
• Fast CBCT showed tumor localization discrepancies for slow and irregular breathing.
• Adaptive acquisition durations may improve CBCT for slow and irregular breathing.

## Graphical abstract

**Figure:**
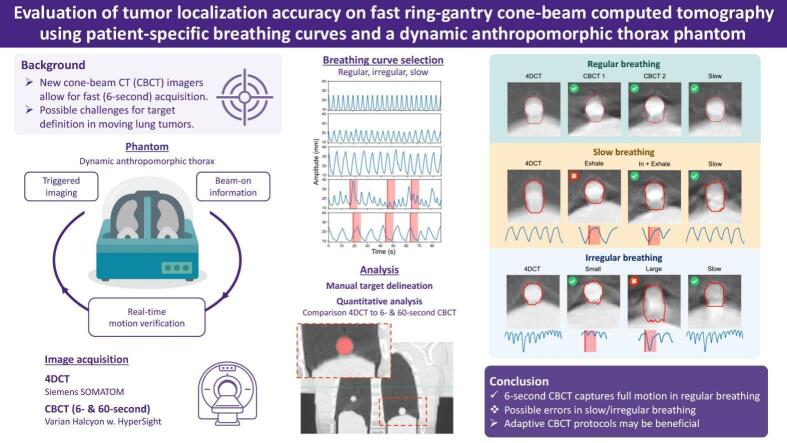

## 1 Introduction

Imaging techniques such as four-dimensional computed tomography (4DCT) are used in radiotherapy to account for tumor movement and to track organs-at-risk (OARs), enabling the definition of the internal target volume (ITV) [1], [2]. This margin is used in treatment planning to compensate for motion uncertainties, and to irradiate the entire tumor position, enabling more precise treatment delivery [3]. At the linear accelerator, additional imaging is performed, using cone-beam CT (CBCT) imaging for positioning verification and assessing if anatomical changes exceed acceptable levels. However, current CBCT images are prone to artifacts, such as motion artifacts, since conventional CBCT scanning takes around 60 s, encompassing multiple breathing cycles.

Recently, integration of CBCT on ring-gantry systems enables fast acquisition (within 6 s), offering improved image quality [4], [5]. It improves CT number accuracies, similar to planning CT imaging, suitable for dose calculation [5], [6], [7], [8], and opens avenues for online adaptive treatment. For regions subject to motion, such as the thorax and abdomen, this adaptive process requires accurate tumor localization and ITV definition. However, 6-second acquisition might pose challenges, e.g. in patients with long breathing periods (>6 s) or amplitude variability. Both situations could result in suboptimal ITV definition, requiring longer 30- or 60-second acquisition.

Zhao et al. [9] showed that for certain combinations of breathing cycle duration and phase, 6-second CBCT acquisition results in discrepancies in tumor shape and localization, compared to the reference average intensity projection (AIP) on 4DCT. Additionally, Koo et al. [10] showed that 6-second CBCT resulted in accurate localization in both regular and moderately irregular breathing patterns. However, highly irregular breathing patterns resulted in discrepancies, with center-of-mass differences larger than 5 mm for both the 6-second and 60-second CBCT acquisition modes.

In the current study, a more realistic dynamic phantom was used compared to the static cylindrical structure with a sphere moved by a piston [9], [10], [11]. A more representative patient anatomy, including intricacies of lung deformation, would allow to fully evaluate the tumor delineation and localization accuracy of fast CBCT acquisition. One of the promising methods of fabricating more realistic phantoms is through 3D-printing, as used in the development of dynamic anthropomorphic thorax and abdominal phantoms [12], [13], [14], [15], [16]. Lustermans et al. (2024) developed a dynamic anthropomorphic thorax phantom, consisting of a 3D-printed static thorax and compressible lungs with realistic internal structures [12]. In addition to realistic phantoms, using patient-derived breathing curves to drive phantom motion allows to evaluate fast CBCT acquisition and target delineation under a range of clinically relevant conditions.

The aim of this study was to evaluate the tumor localization and volumetric accuracy of 6- and 60-second CBCT protocols using a dynamic anthropomorphic thorax phantom, with synchronized imaging capabilities. Both regular and irregular patient-specific, as well as sinusoidal, breathing curves were used to simulate clinically relevant conditions. The target volume and position on the 6-second CBCT was compared to the ground truth delineation on 4DCT, as well as the 60-second CBCT, assessing if 6-second CBCT can accurately capture tumor motion.

## 2 Materials and methods

### 2.1 Thorax phantom

**Fig. 1:**
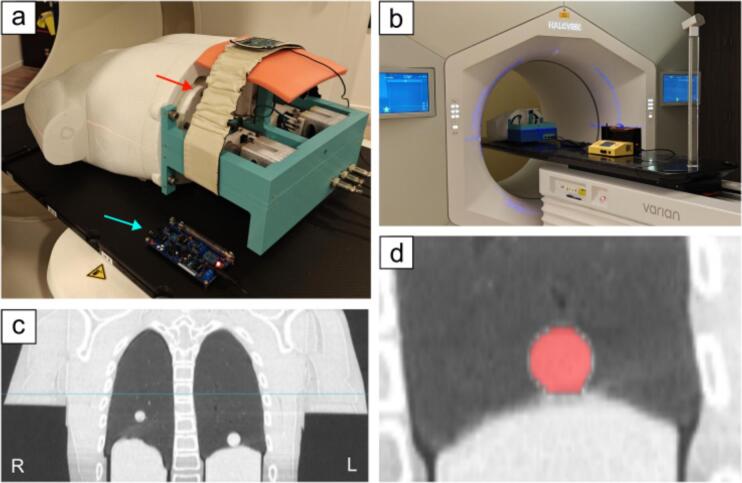
Study workflow. As displayed in Panel a, the phantom was first scanned on a CT scanner, to acquire the average 4DCT reconstruction. The Anzai belt (indicated by the red arrow in Panel a), was used to retrieve the respiratory signal required during 4DCT acquisition from the rigidly moving chest motion platform, which is synchronized to the compression of the lungs. To achieve synchronized image acquisition with the radiation beam, a Geiger counter was used to detect the onset of radiation (indicated by the cyan arrow in Panel a). Next, the phantom was scanned on a Halcyon linear accelerator, as shown in Panel b, to acquire the 6- and 60-second HyperSight CBCT (HS-CBCT) scans. Then, the lower tumor in the left lung was manually delineated in the CT and HS-CBCT scans. Panel c & d show an example of a coronal CT slice with a corresponding tumor segmentation. (For interpretation of the references to colour in this figure legend, the reader is referred to the web version of this article.)

A second-generation version of the in-house developed dynamic anthropomorphic 4DCT thorax phantom, as described in Lustermans et al. [12], was used to represent realistic patient anatomy (Fig. 1). The phantom geometry was modeled based on the extended cardiac-torso mathematical model XCAT [17] and manufactured by fused deposition modeling printing. The phantom was optimized as compared to the previous study [12], by not only distinguishing bone and soft tissue, but further separating soft-tissue into adipose tissue, muscle, heart, and spleen, differentiating them by relative electron density using optimized printing materials and settings. The lungs, containing internal structures, i.e. bronchi and tumors, were compressed using an in-house developed electro-mechanical lung compression system (LCS). Additionally, a separate platform was moved using the chest motion system (CMS), simulating chest movement. Both lungs contained three spherically shaped solid tumors, with a mass density higher than the lung-equivalent tissue, with the lower tumor in the left lung (volume of 3.49 cm^3^) used in this study. A real-time feedback system was used, enabling synchronized phantom movement and showing the current position within the input breathing trace. Furthermore, this system allows to pause phantom motion until radiation is detected, allowing synchronized imaging during specific parts in the breathing curve.

### 2.2 Breathing curves

**Fig. 2:**
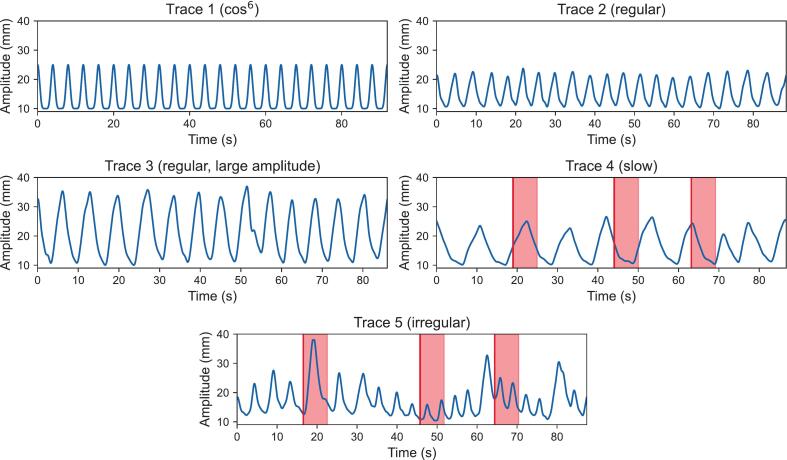
Selected breathing curves with varying breathing period, amplitude and irregularity. Trace 1 is an idealized cos^6^ pattern, and traces 2–5 are derived from real radiotherapy patients with lung cancer. The red line and the shaded region indicate the 6-second scanning regions-of-interest, used for synchronized imaging of the slow and irregular patterns. The breathing traces have a minimum amplitude (initial compression) of 10 mm as this showed optimal lung deformation. The traces as shown in the figure correspond to the signal retrieved from the RPM system and serve as input to the chest motion system (CMS). The signal is inverted for the lung compression system (LCS), so that for exhalation, maximum lung compression represents lower chest position, and vice versa for inhalation as in a patient. (For interpretation of the references to colour in this figure legend, the reader is referred to the web version of this article.)

Five breathing traces were used as input signal to drive the phantom. As a regular reference breathing trace, a ${\text{cos}}^{6}$ pattern [18] with a 4-second period and 15 mm peak-to-valley amplitude was included, represented by Trace 1 (Fig. 2). Four lung cancer patient-derived breathing traces were selected from a cohort of 37 patients undergoing 4DCT acquisition at the Universitätsklinikum Erlangen (Department of Radiation Oncology), based on their characteristics in terms of breathing period, amplitude and irregularity, to capture the full-range of possible scenarios. Irregularities were quantified by the standard deviation of the period (peak-to-peak) and amplitude (peak-to-valley). The breathing curves were acquired during 4DCT acquisition using the RPM Respiratory Gating System (Varian Medical Systems, Inc. Palo Alto, CA, USA). The characteristics of the selected breathing traces, in terms of mean and standard deviation of the period and peak-to-valley amplitude are shown in Table 1. Firstly, a patient was selected displaying regular breathing, represented by Trace 2. Furthermore, patients can display a large amplitude, represented by Trace 3. Both Trace 2 and Trace 3 initially exhibited a slight downward trend. A linear fit was applied to remove this baseline drift, focusing on peak-to-peak variation in these regular traces. Patients may also exhibit breathing periods longer than 6 s, approximately 10% in the cohort of 37 patients, such as Trace 4, and in Werner et al. [19]. Lastly, patients can display large amplitude variability, as represented by Trace 5 with peak-to-valley amplitude ranging from 5 up to 28 mm. The respiratory traces shown in Fig. 2 serve as input to the CMS, where peaks and valleys correspond to inhalation and exhalation, respectively. For the LMS, the input was inverted so that minimum and maximum compression align with inhalation and exhalation, respectively.

**Table 1:** Characteristics of the selected breathing traces, in terms of the mean (± standard deviation) of the period and peak-to-valley amplitude.

**Trace**	**Period (s)**	**Amplitude (mm)**
1	4.0 ± 0.0	15.0 ± 0.0
2	4.4 ± 0.4	11.3 ± 0.8
3	6.2 ± 0.6	23.0 ± 1.2
4	9.7 ± 1.9	9.6 ± 2.1
5	4.2 ± 1.0	10.5 ± 5.1

### 2.3 Image acquisition

For each breathing signal, a reference 4DCT scan of the phantom was acquired on a SOMATOM Definition Drive CT scanner (Siemens Healthineers AG, Forcheim, Germany). The Anzai chest motion belt (Anzai Medical Co. Ltd, Tokyo, Japan) was used to record the respiratory signal from the CMS. The 4DCT scan was reconstructed into eight phases (with 25% increments), using amplitude-binning, as well as an average reconstruction. A clinical thorax imaging protocol was used with a tube voltage of 120 kVp, CTDI_vol,32cm_ of 17.1 mGy and iterative reconstruction (ADMIRE, strength 3). A pixel spacing of 0.977 mm x 0.977 mm, field-of-view of 50 cm and slice thickness of 2 mm were chosen to closely match the CBCT scanning protocol.

CBCT scans of the phantom were acquired on a Halcyon ring gantry linear accelerator, equipped with HyperSight (HS-CBCT) (Varian Medical Systems, Inc. Palo Alto, CA, USA). A clinical imaging protocol was used with a tube voltage of 125 kV and an exposure of 348 mAs and 564 mAs for the fast (6 s)- and slow (60 s)-HS protocol, corresponding to a CTDI_vol_ of 6.6 mGy and 10.7 mGy. These settings for exposure, and thus dose, were higher than the default values for regular imaging, as these images are intended for delineation, and eventually planning. The scanning protocol included a slice thickness of 2 mm, pixel spacing of 1.05 mm x 1.05 mm and iterative iCBCT Acuros reconstruction, with a field-of-view of 53.8 cm.

### 2.4 Experiments and evaluation

To assess the performance of fast HS-CBCT acquisition, multiple scenarios were considered.

The reproducibility of the imaging workflow, including phantom consistency and synchronized image acquisition, was tested by acquiring three fast-HS scans at the same timestamp within irregular breathing Trace 5 (third shaded area), using the real-time feedback system.

The tumor localization of fast HS-CBCT in a ‘clinical workflow’ was evaluated by acquiring fifteen fast-HS scans randomly started in Trace 5. This resembled different imaging moments during treatment fractions, to evaluate the tumor definition accuracy expected throughout the treatment course.

For the regular breathing curves (Trace 1–3), three fast-HS scans were acquired randomly throughout the breathing signal, as well as a slow-HS scan for comparison, to evaluate tumor definition accuracy under regular breathing conditions.

To evaluate maximum expected localization discrepancies in slow and irregular breathing scenarios, three fast-HS scans were acquired for both Trace 4 & Trace 5, at different timestamps representative of maximum motion variability (indicated in red in Fig. 2), using the real-time feedback system. A slow-HS scan was acquired for each trace for comparison.

The HS-CBCT scans were registered to the average 4DCT reconstruction using rigid registration, based on the rigid anatomy of the phantom. Next, 4DCT and CBCT images were randomized and the lower tumor in the left lung was manually delineated by two annotators on the average 4DCT and HS-CBCT scans. This tumor was chosen as a worst case scenario with largest motion and challenging anatomy close to the diaphragm. Delineation was done in the axial, coronal and sagittal views using the brush tool and lung windowing (width and level of 1500 and −600 HU) in the in-house developed software AMIGOpy [20]. The delineations produced by the two annotators were combined by averaging the contours. Details regarding the averaging, as well as inter-observer agreement, can be found in Supplementary Information A (Figs. S1-S3).

The localization accuracy was evaluated using the difference of center-of-mass ($\Delta_{com}$) of the fast- and slow-HS scanned volumes, relative to the average 4DCT reconstruction, in the direction of largest motion, i.e. superior-inferior (SI). The volumetric accuracy was quantified using absolute and relative volume difference (ΔV), as well as volume overlap between the CBCT scans and average 4DCT. The volume overlap was quantified using the Dice Similarity Coefficient (DSC), calculated as per equation (1):

(1) $$DSC=\frac{2|A\cap B|}{\left|A\right|+\left|B\right|}$$

where A and B are 3D-voxelized delineations for which the overlap is quantified.

## 3 Results

The three fast-HS scans for reproducibility testing, automatically acquired at the same timestamp within Trace 5, showed high agreement, with the center-of-mass difference ranging from 0.38 to 1.18 mm and DSC ranging from 0.87 to 0.92 (Table S1). Visually, CBCT2 & 3 shared similar appearance, with CBCT1 showing slightly different appearance and larger delineated tumor volume (Supplementary Fig. S4).

**Fig. 3:**
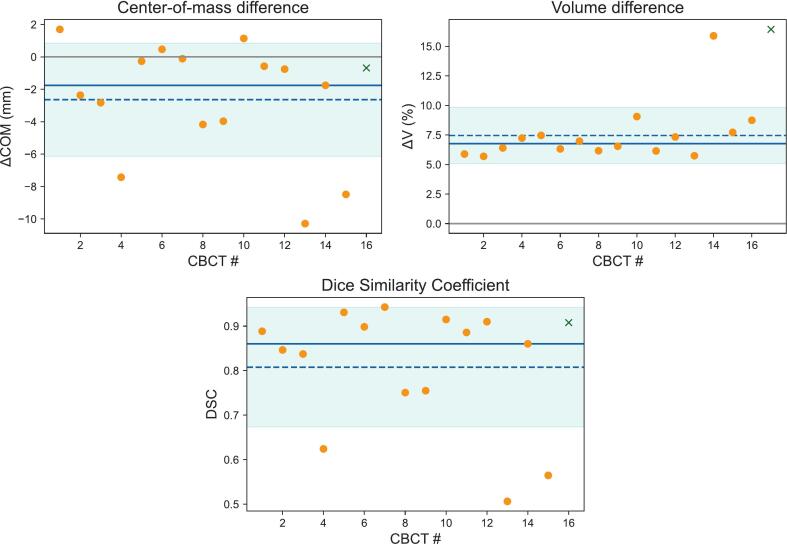
Localization and volumetric accuracy of fifteen tumor delineations (orange datapoints), randomly acquired during Trace 5 (ΔCOM = center-of-mass, ΔV = volume difference, DSC = Dice similarity coefficient). The average delineation (by classifying voxels within at least half of the CBCT masks) is shown by the green cross (16th datapoint). The solid and dotted lines show the median and mean of the datapoints, respectively. The light blue bands indicate the standard deviation of the datapoints. (For interpretation of the references to colour in this figure legend, the reader is referred to the web version of this article.)

The fifteen fast-HS scans, randomly acquired during Trace 5 as in a clinical workflow, showed large variability, both visually (Fig. S5) and quantitatively (Fig. 3, Table S2). The center-of-mass difference ranged between –10.3 and 1.7 mm. The tumor volume was on average overestimated by 29%, up to 170%, compared to the average 4DCT volume. The latter was likely caused by the 6-second acquisition encompassing the large amplitude peak as indicated in Trace 5 (Fig. 2). This resulted in a DSC ranging between 0.51 and 0.94. Averaging the fifteen individual scans, by classifying voxels within at least half of the CBCT masks, showed improved localization and volumetric accuracy, indicated by the green cross in Fig. 3 (16th datapoint), with a mean localization error of −0.7 mm, mean volume difference of 16% and mean DSC of 0.91. Nevertheless, treating the tumor based on the anatomy-of-the-day would result in overdosing of healthy tissue, as on a daily basis the tumor volume is overestimated.

**Fig. 4:**
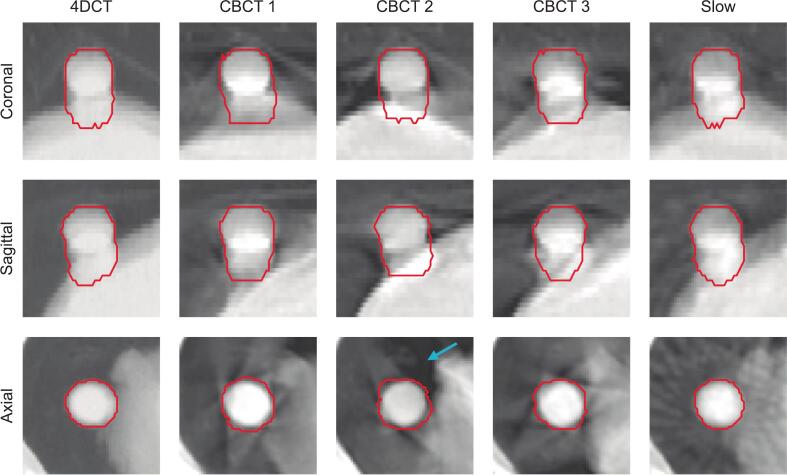
Images and manual contours on the average 4DCT reconstruction, three randomly acquired 6-second HS-CBCT scans (CBCT1-3), as well as the 60-second HS-CBCT scan (Slow), acquired in Trace 1 (cos^6^). The images and contours are displayed in the axial, coronal and sagittal view through the center-of-mass of the contoured tumor. The cyan arrow indicates areas with CT numbers as low as −1000 HU. (For interpretation of the references to colour in this figure legend, the reader is referred to the web version of this article.)

A detailed overview of the metric values for the five selected cases is provided in Table S3, complementing the value ranges discussed below. For Trace 1 (cos^6^), all three fast-HS scans demonstrated accurate target definition, with a DSC of 0.90. In terms of localization and volumetric accuracy, center-of-mass differences ranged from 0.6 mm to 1.4 mm, and volume differences ranged from −2.6 to 7.2%. Patches of unplausible CT numbers, as low as −1000 HU were observed, as indicated by the arrow in Fig. 4. The slow-HS scan also captured the full range of motion with a DSC of 0.94.

For Trace 2 (regular), fast-HS scans showed localization errors ranging from 0.7 to 1.3 mm (Fig. S6). The volume differences ranged from −7.8 up to 2.9%, with a DSC ranging from 0.91 to 0.93. The slow-HS scan demonstrated similar localization and volumetric accuracy with a center-of-mass difference of 0.6 mm, volume difference of −6.5% and DSC of 0.95.

The fast-HS scans for Trace 3 (regular, large amplitude) exhibited considerable variability in both tumor localization and volume estimation. Volume differences ranged from 9 up to 25%, with center-of-mass differences ranging from 0.5 to 2.1 mm, compared to the 4DCT reference. Combined, volume and localization discrepancies resulted in a DSC ranging from 0.83 to 0.88. Blurring and artifacts were prominent in the fast-HS scans, distorting the spherical target shape (Fig. S7). The slow-HS scan provided improved results, with a localization error of −0.3 mm, volume difference of 6%, and DSC of 0.94.

**Fig. 5:**
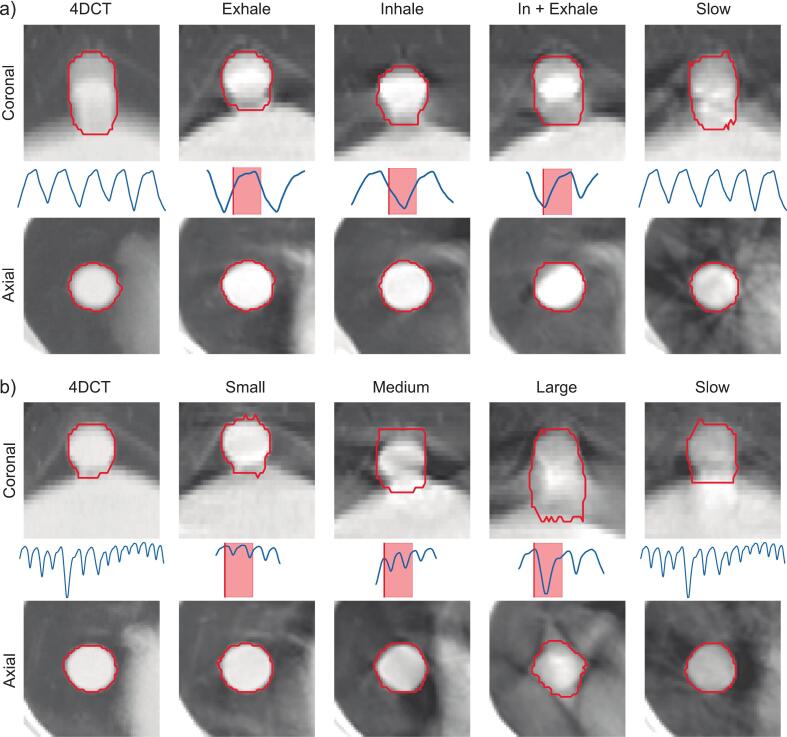
Images and manual contours on the average 4DCT reconstruction, three 6-second HS-CBCT scans, as well as the 60-second HS-CBCT scan (Slow) of the slow (a) and irregular (b) breathing trace. The 6-second HS-CBCT scans were timed at different timestamps (shaded red area) corresponding to Exhale-only, Inhale-only & Inhale-to-exhale for slow breathing (a) and Small, Medium & Large magnitude of breathing motion for irregular breathing (b). The images and contours are displayed in the axial and coronal view through the center-of-mass of the contoured tumor. The traces indicate the input provided to the lung compression system and indicate the extent of lung compression. (For interpretation of the references to colour in this figure legend, the reader is referred to the web version of this article.)

For slow breathing (Trace 4), the fast-HS scan acquired during exhalation-only showed large localization discrepancy, with a center-of-mass difference of 5.2 mm (Fig. 5a). Volumetrically, both the exhalation- and inhalation-only scans resulted in a volume underestimation of −31%, with a DSC of 0.76 and 0.79. The fast-HS scan acquired from max-inhalation to max-exhalation yielded improved volume definition with a volume difference of −12%, and DSC of 0.89. The slow-HS scan showed comparable results to the latter, with a volume difference of −13% and DSC of 0.91.

For irregular amplitude breathing (Trace 5), the three fast-HS scans showed high variability, both in terms of localization and volumetric accuracy (Fig. 5b). The small, medium and large amplitude corresponded to center-of-mass differences of 1.8, −4.0 and −10.3 mm and volume overestimation of 6, 38 and 119%, resulting in a DSC of 0.89, 0.76 and 0.57, respectively. The slow-HS scan demonstrated best localization, with a localization error of −2 mm, but also volume overestimation of 24%, resulting in a DSC of 0.86.

## 4 Discussion

This study evaluated the accuracy of lung tumor localization and volumetric assessment in HS-CBCT scans under various patient-specific respiratory scenarios. This was conducted using a dynamic anthropomorphic thorax phantom, equipped with a feedback-control system that triggers motion at predefined points along the cycle. Results demonstrated that, for regular breathing patterns with cycle durations below 6 s, the full extent of target motion could be captured. However, for longer breathing cycles or irregular respiratory patterns, motion range detection was insufficient depending on scan location, resulting in center-of-mass and volume differences up to 10.3 mm and 119%.

For the simulated cos^6^, as well as the regular patient-derived breathing trace, both fast- and slow-HS scan protocols were able to capture the full target motion. Nevertheless, significant motion artifacts around the tumor, with CT numbers as low as –1000 HU, were observed in both fast and slow HS-CBCT scans (Fig. 4). The fast-HS scans for the large amplitude breathing curve showed more discrepancies, primarily attributed to significant blurring and artifacts (Fig. S7). Increasing the imaging dose, using a pelvic imaging protocol, did not show to impact the presence and appearance of artefacts (Fig. S8). As a result, the spherical shape of the target was distorted, also shown by Oliver et al. [11]. The imaging plane in which the tumor shape was most distinguishable varied considerably, possibly explained by acquisition of different projection angles at different phases of target motion.

For patients with a breathing period exceeding the 6-second scan duration, capturing the full motion range is not guaranteed (Fig. 5a). Notable localization and volume discrepancies were found (Table S3), consistent with findings by Zhao et al. [9]. They showed that tumor position agreement, between the ITV derived from the fast-HS scan and the average intensity projection on 4DCT, is highly dependent on the motion phase during CBCT acquisition. Similarly, for patients who show large amplitude variability and cannot be coached to breathe more regularly during treatment, the fast-HS can either under- or overestimate tumor position and volume, resulting in inaccurate dosing. Therefore, monitoring respiration during imaging and treatment, or using breath-hold techniques, may be desirable, particularly given daily breathing pattern variations [2].

Although the phantom is already substantially more realistic, improvements are to be made in terms of expandable chest with moving ribs, including tumor motion in multiple directions. Furthermore, this set-up would enable simultaneous evaluation of tumor delineation accuracy as well as overall image quality, as moving ribs are expected to cause more pronounced streaking artifacts, impacting dose calculation. Even though the average CT was used as reference in this study, as it most resembles CBCT, other methods such as an ITV approach based on the min–max breathing phases may be more suitable. Nevertheless, this is not expected to impact the findings regarding the challenges of 6-second CBCT in the case of slow and irregular breathing. Next, while the inter-observer agreement between the delineations by two annotators was high and similar for 4DCT and CBCT, indicated by a median DSC of 0.89 and 0.88 as shown in Fig. S3, this was a source of uncertainty not further evaluated in the current study. Another limitation of this study is that only 6- and 60-second scan durations were evaluated, currently available in our clinic. Intermediate acquisition durations (e.g., 15 s) could potentially offer a balance between motion range capture and motion-induced artefacts reduction. Additionally, although a representative selection of breathing scenarios was evaluated, the dataset of evaluated respiratory traces could be increased. Furthermore, the findings in this study could be validated for other anatomical regions subject to motion, such as the abdomen [14], [21]. Another future interest, would be the evaluation of tumor motion based on fast 4D-CBCT [22]. Lastly, the clinical relevance should be evaluated regarding clinical margins and assessing dose calculations.

The results in this study suggest that for regular breathing patterns, fast-HS scans might be able to capture the full range of target motion sufficiently. However, localization and volumetric discrepancies can occur when a patient exhibits slow (>6 s) or irregular breathing. Therefore, careful consideration is needed when selecting protocols for lung tumor cases in both online and offline adaptive radiotherapy.

## Data availability

The datasets generated during the current study, including imaging data, manual delineations and anonymized patient breathing traces, are available on Zenodo (version v1) at https://doi.org/10.5281/zenodo.17256015 [23].

## CRediT authorship contribution statement

**Lars H.B.A. Daenen:** Methodology, Software, Formal analysis, Investigation, Data curation, Writing – original draft, Visualization. **Didier Lustermans:** Writing – review & editing, Methodology, Investigation, Conceptualization. **Tim H.A. Stassen:** Writing – review & editing, Resources. **Juliane Szkitsak:** Writing – review & editing, Resources. **Roua Abdulrahim:** Writing – review & editing, Resources. **Jo Goossens:** Resources. **Gina Rishmawi:** Methodology. **Richard Canters:** Writing – review & editing. **Ilaria Rinaldi:** Writing – review & editing. **Frank Verhaegen:** Writing – review & editing, Supervision. **Gabriel Paiva Fonseca:** Writing – review & editing, Supervision, Methodology, Conceptualization.

## Declaration of competing interest

The authors declare that they have no known competing financial interests or personal relationships that could have appeared to influence the work reported in this paper.

## References

[1] Brandner E.D., Chetty I.J., Giaddui T.G., Xiao Y., Saiful H.M. (2017). Motion management strategies and technical issues associated with stereotactic body radiotherapy of thoracic and upper abdominal tumors: a review from NRG oncology. Med Phys.

[2] Dhont J., Vandemeulebroucke J., Burghelea M., Poels K., Depuydt T., Van Den Begin R. (2018). The long- and short-term variability of breathing induced tumor motion in lung and liver over the course of a radiotherapy treatment. Radiother Oncol.

[3] Yamamoto T., Kabus S., Bal M., Keall P., Benedict S., Daly M. (2016). The first patient treatment of computed tomography ventilation functional image-guided radiotherapy for lung cancer. Radiother Oncol.

[4] Lustermans D., Fonseca G.P., Taasti V.T., van de Schoot A., Petit S., van Elmpt W. (2024). Image quality evaluation of a new high-performance ring-gantry cone-beam computed tomography imager. Phys Med Biol.

[5] Robar J.L., Cherpak A., MacDonald R.L., Yashayaeva A., McAloney D., McMaster N. (2024). Novel Technology allowing Cone Beam Computed Tomography in 6 seconds: a Patient Study of Comparative image Quality. Pract Radiat Oncol.

[6] Kunnen B., van de Schoot A.J.A.J., Fremeijer K.P., Nicolai-Koornneef E.M., Offereins-van Harten K., Sluijter J.H. (2024). The added value of a new high-performance ring-gantry CBCT imaging system for prostate cancer patients. Radiother Oncol.

[7] Bogowicz M., Lustermans D., Taasti V.T., Hazelaar C., Verhaegen F., Fonseca G.P. (2024). Evaluation of a cone-beam computed tomography system calibrated for accurate radiotherapy dose calculation. Phys Imaging Radiat Oncol.

[8] Sijtsema N.D., Penninkhof J.J., van de Schoot A.J.A.J., Kunnen B., Sluijter J.H., van de Pol M. (2025). Dose calculation accuracy of a new high-performance ring-gantry CBCT imaging system for prostate and lung cancer patients. Radiother Oncol.

[9] Zhao H, Nelson G, Sarkar V, Oare C, Szegedi M, St. James S, et al. Comprehensive Image Quality Evaluation and Motion Phantom Studies of an Ultra-Fast (6-Second) Cone-Beam Computed Tomography Imaging System on a Ring Gantry Linear Accelerator. Adv Radiat Oncol 2025;10:101681. https://doi.org/10.1016/j.adro.2024.101681. 10.1016/j.adro.2024.101681 PMC11665466 39717196

[10] Koo J, Redler G, Semenenko V, Rosenberg SA, Keit E, Andreozzi JM. Localization accuracy of 6-second CBCT for lung IGRT with various breathing patterns. J Appl Clin Med Phys n.d.;26:e70130. https://doi.org/10.1002/acm2.70130. 10.1002/acm2.70130 PMC12256656 40439857

[11] Oliver P.A.K., Montgomery L., Granville D.A. (2025). Cone beam computed tomography in 6- and 60-second acquisitions: implications for adaptive radiotherapy when respiratory motion is present. Biomed Phys Eng Express.

[12] Lustermans D., Abdulrahim R., Taasti V.T., Szkitsak J., Švėgždaitė E., Clarkin S. (2024). Development of a novel 3D-printed dynamic anthropomorphic thorax phantom for evaluation of four-dimensional computed tomography. Phys Imaging Radiat Oncol.

[13] Stengl C., Panow K., Arbes E., Muñoz I.D., Christensen J.B., Neelsen C. (2023). A phantom to simulate organ motion and its effect on dose distribution in carbon ion therapy for pancreatic cancer. Phys Med Biol.

[14] Bakhtiari Moghaddam A., Runz A., Augusto R.F., Echner G., Johnen W., Gabriel R. (2025). A dynamic anthropomorphic phantom for end-to-end testing in image- and surface-guided adaptive radiotherapy. Med Phys.

[15] Abdollahi S., Mowlavi A.A., Yazdi M.H.H., Ceberg S., Aznar M.C., Tabrizi F.V. (2024). Dynamic anthropomorphic thorax phantom for quality assurance of motion management in radiotherapy. Phys Imaging Radiat Oncol.

[16] Im J.Y., Micah N., Perkins A.E., Mei K., Geagan M., Roshkovan L. (2025). PixelPrint 4D : a 3D Printing Method of Fabricating Patient-specific Deformable CT Phantoms for respiratory Motion applications. Invest Radiol.

[17] Segars W.P., Mahesh M., Beck T.J., Frey E.C., Tsui B.M.W. (2008). Realistic CT simulation using the 4D XCAT phantom. Med Phys.

[18] Lujan A.E., Balter J.M., Ten Haken R.K. (2003). A method for incorporating organ motion due to breathing into 3D dose calculations in the liver: Sensitivity to variations in motion. Med Phys.

[19] Werner R., Sentker T., Madesta F., Gauer T., Hofmann C. (2019). Intelligent 4D CT sequence scanning (i4DCT): Concept and performance evaluation. Med Phys.

[20] Paiva Fonseca G, Daenen L. PhysicsResearch/AMIGOpy: AMIGOpy v0.7 2026. https://doi.org/10.5281/zenodo.20313209.

[21] Stengl C., Christensen J.B., Muñoz I.D., Neuholz A., Brons S., Yukihara E.G. (2025). Dose assessment in moving targets and organs at risk during carbon ion therapy for pancreatic cancer with respiratory gating. Phys Imaging Radiat Oncol.

[22] Gardner M., Dillon O., Reynolds T., Kipritidis J., Bazalova-Carter M., Byrne H. (2025). Evaluation of 4D cone-beam CT reconstruction methods for lung images acquired using rapid cone-beam CT acquisition: a phantom study. Phys Med Biol.

[23] Daenen L, Lustermans D, Stassen THA, Szkitsak J, Abdulrahim R, Goossens J, et al. 4D and cone-beam CT scans of a dynamic anthropomorphic thorax phantom and patient-specific breathing traces 2025. https://doi.org/10.5281/zenodo.17256015.

