# Supplementary material for: Evaluation of tumor localization accuracy on fast ring-gantry cone-beam computed tomography using patient-specific breathing curves and a dynamic anthropomorphic thorax phantom
Source: Phys Imaging Radiat Oncol. 2026 Jun 3;39:101014. doi: 10.1016/j.phro.2026.101014 (PMC13264287; doi:10.1016/j.phro.2026.101014)
Supplement: Supplementary Data 1 [file mmc1.pdf]

Supplementary material

A. Unification of annotation delineations

For the main analysis, the delineations by the two annotators were combined into a single mask, using a surface distance approach as illustrated in Figure S1. For each of the delineations, the Euclidian distance from the boundary was calculated, taking the voxel spacing into account. A distance map was generated, with the distances inside and outside the masks set to negative and positive values, respectively. Next, the resulting distance maps of the two delineations were added voxel-wise and voxels with a value below zero where classified as foreground. This approach sets voxels to foreground when, either within both masks (both distances negative) or when the local inner boundary is closer than the local outer boundary. As shown in Figure S2, the average contours are positioned in between the contours by the two annotators.

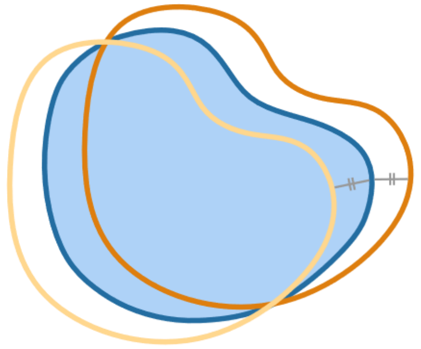

Figure S1: Schematic illustration of the averaging process of the delineations by two annotators.

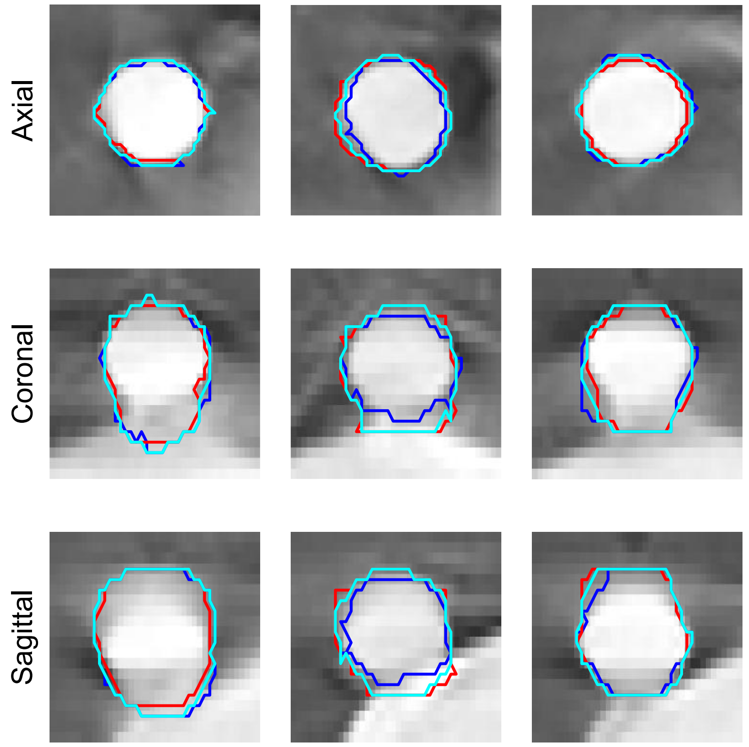

Figure S2: Averaging result of three HS-CBCT images. The delineations by individual annotators correspond to red and blue contours, with the average corresponding to the cyan contour. The images and contours are displayed in the axial, coronal and sagittal planes through the center-of-mass of the contoured tumor.

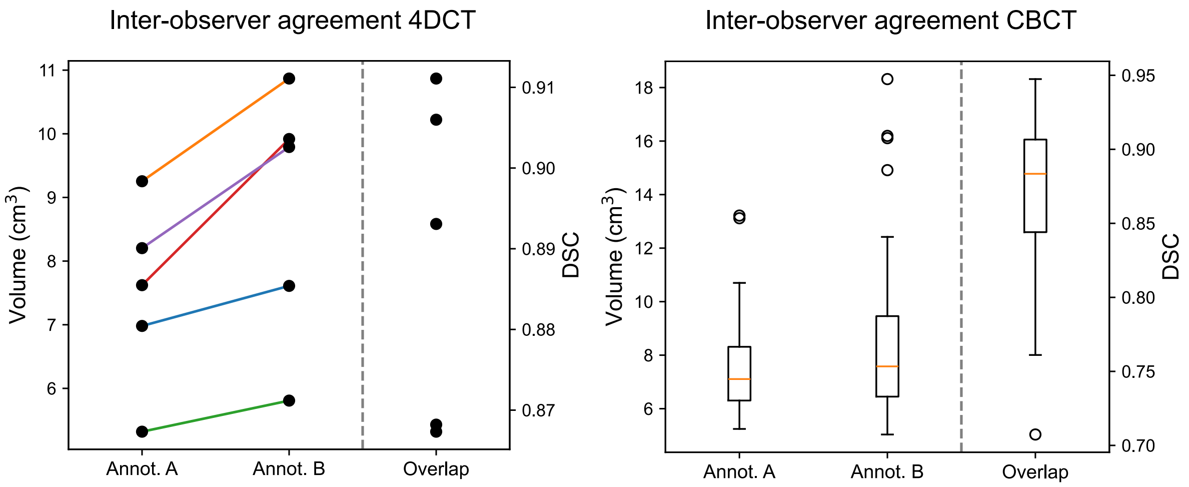

Figure S3: Inter-observer agreement between two annotators for the 4DCT and CBCT scans. The volumes of the delineations created by two annotators are shown in the left panels. The overlap between corresponding delineations, quantified by the Dice Similarity Coefficient (DSC), is shown in the right panels. A dot- and boxplot was used because of different number of samples for the 4DCT (n=5) and CBCT (n=38) analysis.

Figure S3 shows the distribution of the delineation volumes created by the two annotators, as well as the overlap between corresponding delineations in terms of the Dice Similarity Coefficient (DSC). The median DSC was 0.89 and 0.88 for the 4DCT and CBCT scans, respectively.

B. Extended results of reproducibility evaluation

Table S1: Center-of-mass difference (Δ_{com}), absolute & relative volume difference (ΔV), and Dice Similarity Coefficient (DSC) for the reproducibility scans. The metrics were calculated pairwise between the three 6-second HS-CBCT scans. The combinations resulting in a DSC >0.85 were highlighted.

	Localization	***Volume***		***Overlap***
	Δ_{com}(mm)	ΔV (cm^3)	ΔV (%)	DSC
**1 vs. 2**	0.38	-0.99	-12.3	0.89
**1 vs. 3**	1.18	-1.42	-17.6	0.87
**3 vs. 2**	0.81	-0.43	-6.0	0.92
**Mean abs.**	0.79	0.94	12.0	0.89
**STD**	0.33	0.40	4.7	0.02

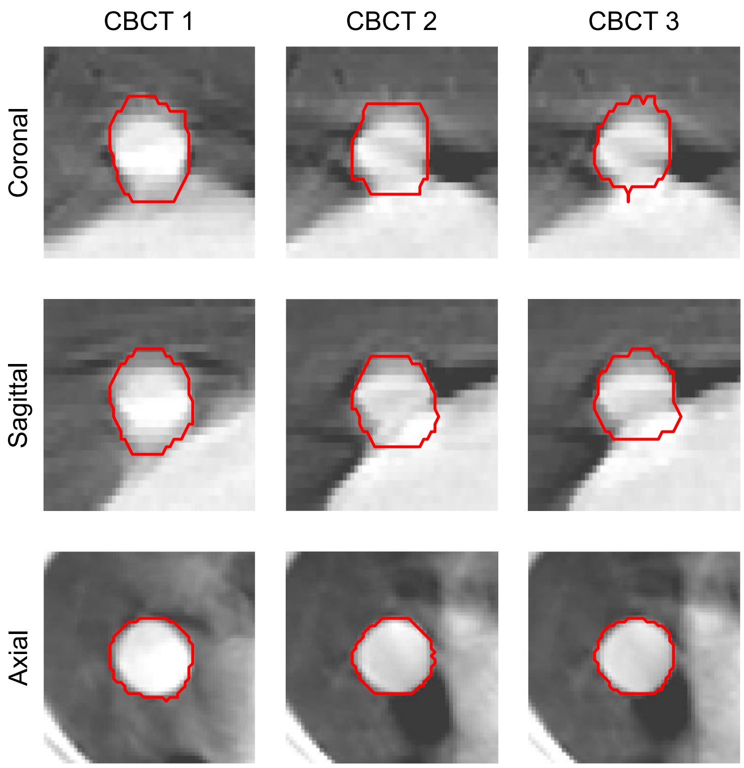

Figure S4: Axial, coronal and sagittal views of the three 6-second HS-CBCT scans acquired at the identical timestamp within **Trace 5**. The images and contours are displayed in the axial, coronal and sagittal planes through the center-of-mass of the contoured tumor.

C. Extended results of clinical workflow evaluation

Table S2: Center-of-mass difference (Δ_{com}), absolute and relative volume difference (ΔV), and Dice Similarity Coefficient (DSC) for the 15 random 6-second HS-CBCT scans in Trace 5 (Clinical workflow). The metrics were calculated for the CBCT scans, relative to the average 4DCT reconstruction. The CBCT scans resulting in a DSC >0.85 were highlighted.

Clinical workflow

	Localization	***Volume***		***Overlap***
	Δ_{com}(mm)	ΔV (cm^3)	ΔV (%)	DSC
**1**	1.70	-0.19	-3.3	0.89
**2**	-2.36	0.52	8.8	0.85
**3**	-2.83	1.35	23.0	0.84
**4**	-7.42	1.58	26.8	0.62
**5**	-0.27	0.43	7.4	0.93
**6**	0.47	1.09	18.5	0.90
**7**	-0.11	0.28	4.7	0.94
**8**	-4.17	0.66	11.2	0.75
**9**	-3.97	3.17	53.9	0.75
**10**	1.14	0.26	4.4	0.91
**11**	-0.57	1.44	24.5	0.89
**12**	-0.75	-0.14	-2.4	0.91
**13**	-10.28	10.00	170.0	0.51
**14**	-1.75	1.84	31.2	0.86
**15**	-8.48	2.86	48.6	0.56
**Mean abs.**	3.09	1.72	29.3	0.81
**±STD**	3.11	2.39	40.7	0.13

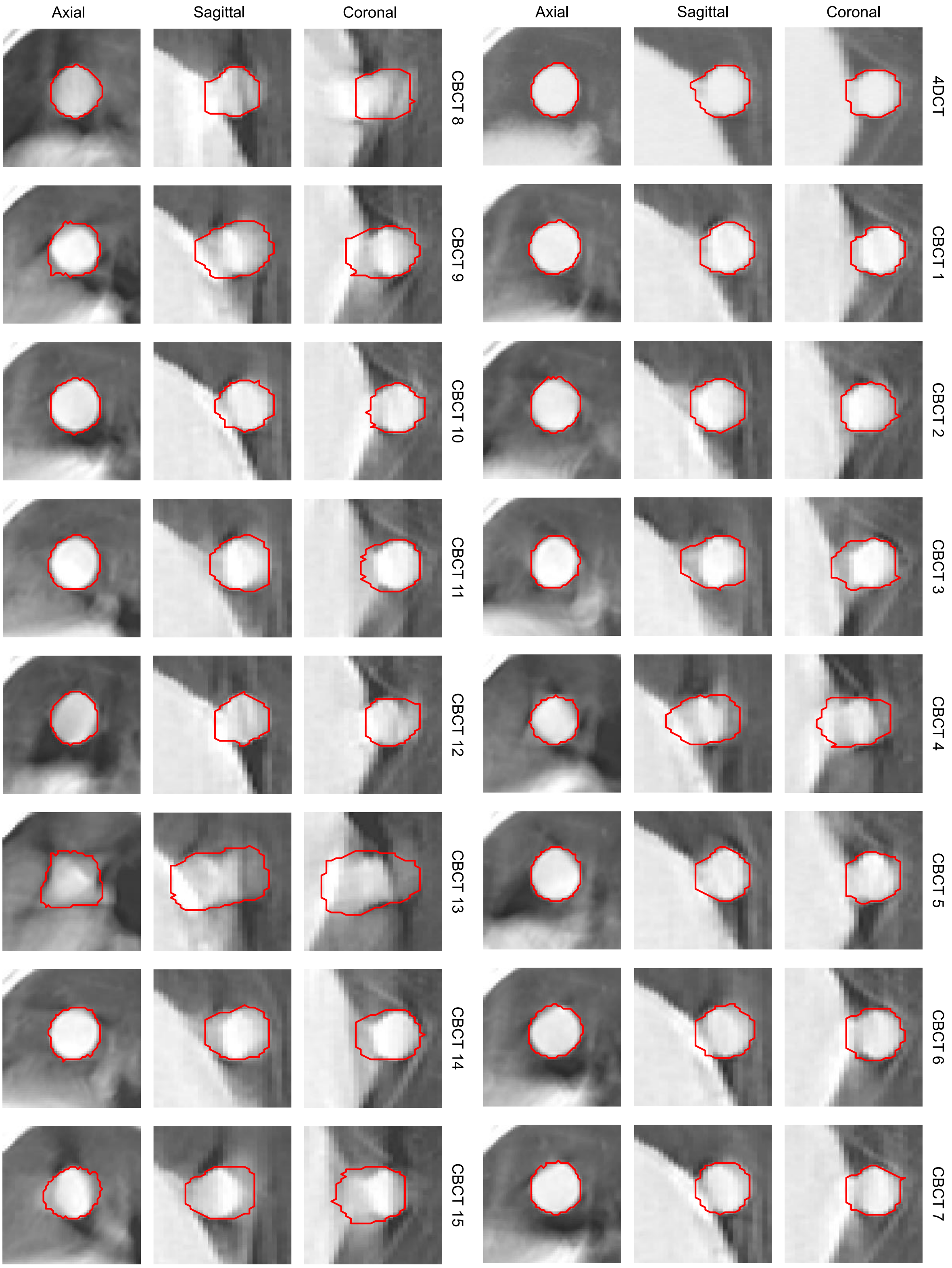

Figure S5: The axial, coronal and sagittal planes of the fifteen randomly acquired 6-second HS-CBCT scans in **Trace 5 (irregular amplitude)**. The images and contours are displayed in the axial, coronal and sagittal planes through the center-of-mass of the contoured tumor.

D. Extended results of selected traces

Table S3: Center-of-mass difference (Δ_{com}), absolute and relative volume difference (ΔV), and Dice Similarity Coefficient (DSC) for the five breathing traces. The metrics were calculated for the HS-CBCT scans, relative to the average 4DCT reconstruction. The CBCT scans resulting in a DSC >0.85 were highlighted.

a) Trace 1: Regular ($\cos^6$)

	Localization	*Volume*		*Overlap*
	Δ_{com}(mm)	ΔV (cm^3)	ΔV (%)	DSC
**1**	0.62	0.67	7.19	0.90
**2**	1.36	0.25	2.63	0.90
**3**	1.25	-0.24	-2.57	0.90
**Mean abs.**	1.07	0.39	4.13	0.90
**STD**	0.33	0.20	2.16	0.00
**Slow**	0.76	-0.57	-6.09	0.94

b) Trace 2: Regular (patient-derived)

	Localization	*Volume*		*Overlap*
	Δ_{com}(mm)	ΔV (cm^3)	ΔV (%)	DSC
**1**	1.28	-0.27	-3.51	0.91
**2**	0.71	0.22	2.87	0.93
**3**	1.27	-0.61	-7.83	0.91
**Mean abs.**	1.09	0.37	4.74	0.92
**STD**	0.27	0.17	2.20	0.01
**Slow**	0.60	-0.50	-6.45	0.95

c) Trace 3: Large amplitude

	Localization	*Volume*		*Overlap*
	Δ_{com}(mm)	ΔV (cm^3)	ΔV (%)	DSC
**1**	1.18	1.00	9.4	0.84
**2**	2.11	2.70	25.2	0.83
**3**	0.53	1.54	14.4	0.88
**Mean abs.**	1.27	1.75	16.3	0.85
**STD**	0.65	0.71	6.6	0.02
**Slow**	-0.26	0.62	5.8	0.94

d) Trace 4: Long period

	Localization	*Volume*		*Overlap*
	Δ_{com}(mm)	ΔV (cm^3)	ΔV (%)	DSC
**Exhale-only**	5.17	-2.97	-31.24	0.76
**Inhale-only**	-0.71	-2.97	-31.16	0.79
**In & exhale**	1.49	-1.10	-11.56	0.89
**Slow**	1.11	-1.23	-12.97	0.91

e) Trace 5: Irregular amplitude

	Localization	*Volume*		*Overlap*
	Δ_{com}(mm)	ΔV (cm^3)	ΔV (%)	DSC
**Small**	1.83	0.35	5.9	0.89
**Medium**	-4.00	2.24	38.2	0.76
**Large**	-10.34	6.99	118.8	0.57
**Slow**	-1.96	1.41	24.0	0.86

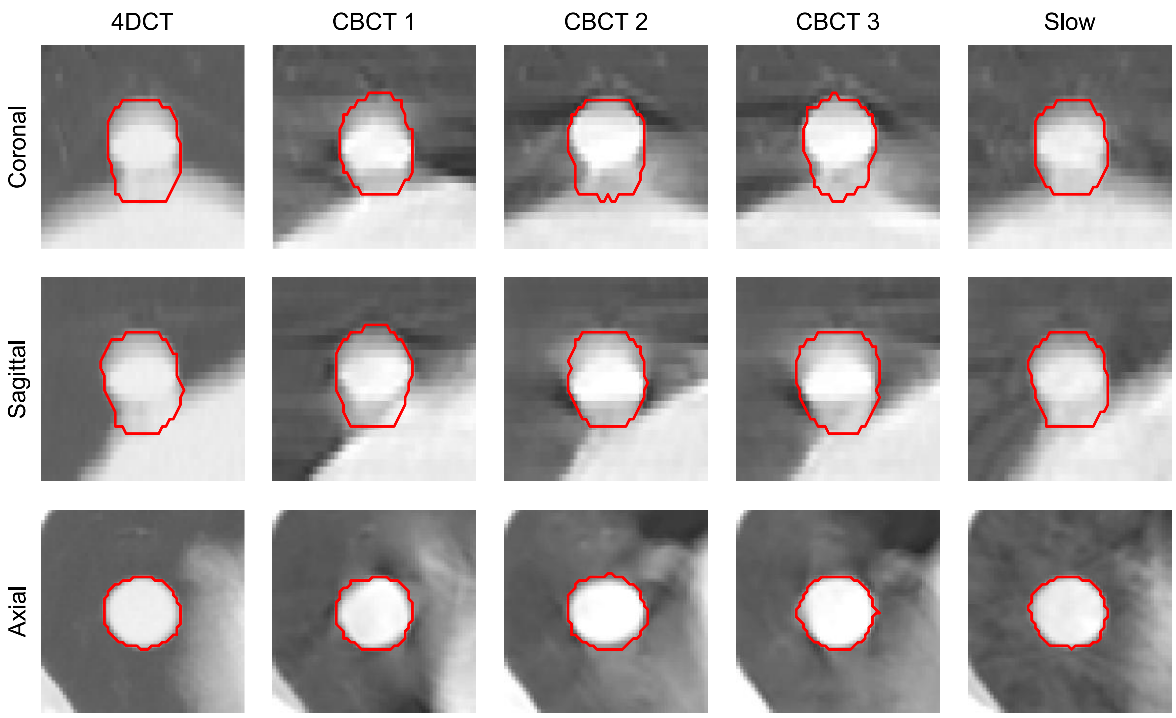

Figure S6: Axial, coronal and sagittal views of the average *4DCT* reconstruction, three 6-second (*CBCT1-3*) and 60-second (*Slow*) HS-CBCT scans, acquired randomly in **Trace 2 (regular patient-derived trace).** The images and contours are displayed in the axial, coronal and sagittal planes through the center-of-mass of the contoured tumor.

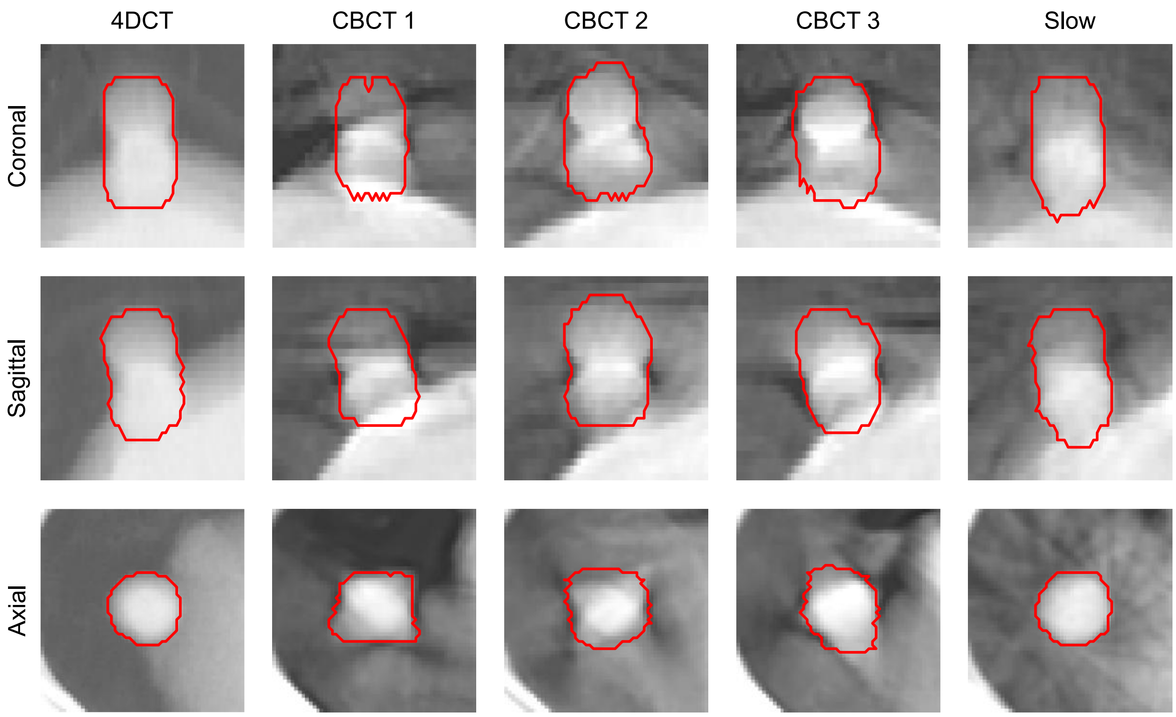

Figure S7: Axial, coronal and sagittal views of the average *4DCT* reconstruction, three 6-second (*CBCT 1-3*) and 60-second (*Slow*) HS-scans, acquired randomly in **Trace 3 (large amplitude)**. The images and contours are displayed in the axial, coronal and sagittal planes through the center-of-mass of the contoured tumor.

E. Dose Comparison

In an online adaptive radiotherapy setting, the cone-beam CT images would be intended for delineation and dose calculation. As this requires optimal image quality, the mAs was increased to the maximum possible values in the 6-second and 60-second thorax protocols. To evaluate the effect of the dose on tumor conspicuity and artefacts, a set of scans was also acquired using a pelvic protocol, which allows the mAs to be increased even further. However, increasing the dose did not show to impact the tumor appearance, presence of artefacts and patches with -1000 HU, as shown in Figure S7.

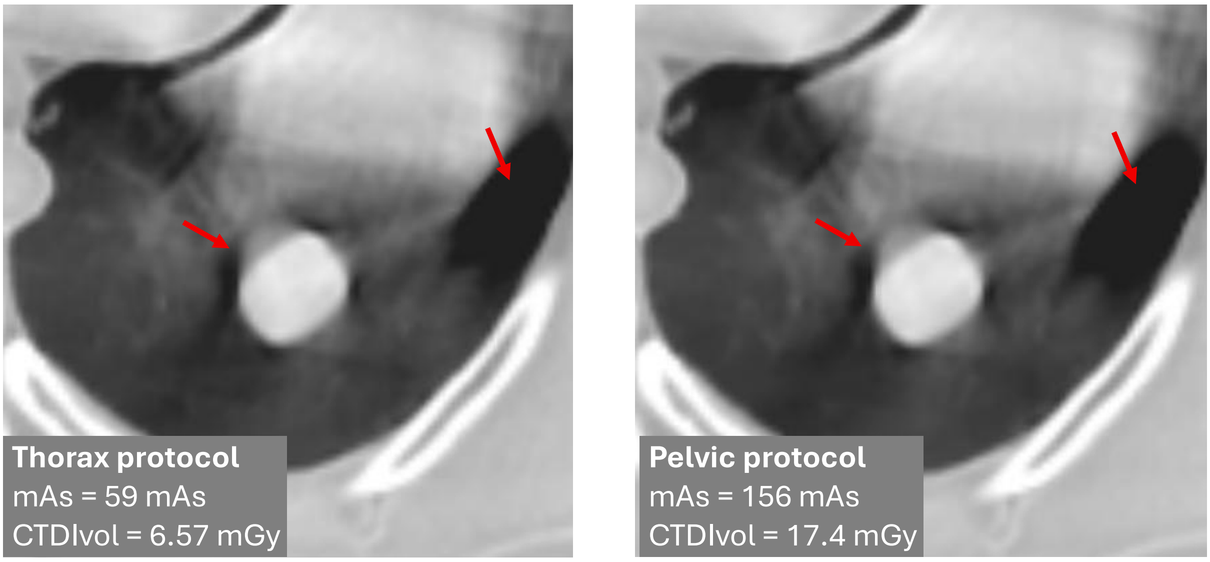

Figure S8: Influence of imaging dose on tumor conspicuity and presence of artefacts, using two different 6-second cone-beam CT scanning protocols.
